# On the predictibility of A-minor motifs from their local contexts

**DOI:** 10.1080/15476286.2022.2144611

**Published:** 2022-11-16

**Authors:** Coline Gianfrotta, Vladimir Reinharz, Olivier Lespinet, Dominique Barth, Alain Denise

**Affiliations:** a Données et Algorithmes pour une Ville Intelligente et Durable (DAVID), Université de Versailles Saint-Quentin-en-Yvelines, Université Paris-Saclay, Versailles, France; b Laboratoire Interdisciplinaire des Sciences du Numérique (LISN), Université Paris-Saclay, CNRS, Orsay, France; c Department of Computer Science, Université du Québec à Montréal, Québec, Canada; d Institute for Integrative Biology of the Cell (I2BC), Université Paris-Saclay, CEA, CNRS, Gif-sur-Yvette, France

**Keywords:** RNA 3D structures, RNA folding, A-minor motif, graph algorithms, clustering

## Abstract

This study investigates the importance of the structural context in the formation of a type I/II A-minor motif. This very frequent structural motif has been shown to be important in the spatial folding of RNA molecules. We developed an automated method to classify A-minor motif occurrences according to their 3D context similarities, and we used a graph approach to represent both the structural A-minor motif occurrences and their classes at different scales. This approach leads us to uncover new subclasses of A-minor motif occurrences according to their local 3D similarities. The majority of classes are composed of homologous occurrences, but some of them are composed of non-homologous occurrences. The different classifications we obtain allow us to better understand the importance of the context in the formation of A-minor motifs. In a second step, we investigate how much knowledge of the context around an A-minor motif can help to infer its presence (and position). More specifically, we want to determine what kind of information, contained in the structural context, can be useful to characterize and predict A-minor motifs. We show that, for some A-minor motifs, the topology combined with a sequence signal is sufficient to predict the presence and the position of an A-minor motif occurrence. In most other cases, these signals are not sufficient for predicting the A-minor motif, however we show that they are good signals for this purpose. All the classification and prediction pipelines rely on automated processes, for which we describe the underlying algorithms and parameters.

Our knowledge of RNA molecules is expanding constantly. New non-coding RNAs are regularly discovered [1] and the function of some of them is not yet elucidated. The functions already known for non-coding RNAs are very diverse and numerous, such as the regulation of gene expression [2,3], RNA modification [4] or protection of the cell [5,6]. The function of an RNA molecule is strongly related to the three-dimensional structure it adopts. This is why many works for more than 40 years have been dedicated to try to predict the 3D structure of an RNA molecule from its primary sequence, which remains an open problem in many aspects.

Besides well-known canonical interactions, lower energy interactions that do not belong to a secondary structure play an important role in the formation of the three-dimensional structure of an RNA molecule [7,8]. They are much more difficult to predict than the canonical interactions.

They are often organized in recurrent sets, which form substructures folding in an identical or almost identical way [9,10]. These small substructures appearing recurrently at various locations in different RNA molecules are called *structural motifs* or *modules*. These modules play a fundamental role in the basic architecture of RNA structures, and their detection can thus drastically increase the quality of predictions of 3D RNA structures.

Structural motifs appearing within a secondary structure loop have been extensively studied, and listed in regularly updated databases [11,12]. Many methods to detect them in tertiary structures have been developed. Some of these methods search for modules by their geometry [13–18], while others use graph theory algorithms by modelling the interactions within these modules, and sometimes using the associated sequences [7,19–22]. In particular, these methods allow the detection of known structural motifs in new structures.

In contrast, structural motifs binding several distinct secondary structure elements have been much less studied from this point of view. The only method, to the best of our knowledge, to exhaustively identify this type of motif is a graph-based method described in [23,24].

Moreover, long-range motifs still remain unpredictable by the methods discussed above. This is in particular the case of the A-minor motif [25,26]. The type I/II A-minor motif, which will be the focus of our study, is an assembly of two consecutive nucleotides (often A) interacting by non-canonical interactions with two consecutive canonical pairs. Present in many families of non-coding RNAs (ribosomal RNAs, transfer RNAs, riboswitches, introns …), this motif represents more than 80% of the non-canonical interactions binding several secondary structure elements [23]. It has been shown to be important in the spatial folding of RNA molecules, as well as in cellular mechanisms such as codon-anticodon recognition during translation [25]. Its prediction is thus an interest in itself, despite the difficulties represented by the involvement of non-canonical interactions, the often large distance on the sequence between the two secondary structure elements, and the lack of sequence signature.

A recent study [27] focused on the classification and prediction of this particular structural motif, by a machine learning method.

This method succeeds in predicting some particular A-minor motif occurrences, appearing jointly with a pseudoknot between secondary structure elements close to each other on the sequence. The other occurrences of A-minor motifs remain unpredictable.

These difficulties are the reasons why our research concerns this particular structural motif.

As said before, we focus here on type I/II A-minor motifs. Usually, these motifs require two As at the positions interacting with the cWW base pairs. Meanwhile, as in [23], we consider a less constrained definition A-minor type I/II motif, where either A can be replaced by another nucleotide.

The main purpose of our study is to understand the relationships between type I/II A-minor motifs and their structural contexts. More specifically, we want to determine what kind of information, contained in the structural context, can be useful to characterize and predict the presence and the position of A-minor motifs. We consider here a structural context at two scales: on the one hand, the 3D substructure of the molecule that surrounds the motif, up to a certain distance, which we call *3D structural context*, and on the other hand, the graph composed of the canonical and non-canonical interactions appearing in this substructure. We call it the *topological structural context*.

To answer our question, we first define an automated way to classify A-minor motifs by comparing their 3D contexts (partially or totally). The different classifications we obtain, depending on the parts of the 3D structural context taken into account, allow us to make some reflections on the importance of the context. In a second step, we study the possibility of predicting the presence of A-minor motifs by assuming that the 3D context is not known, but we only know the topological context and possibly sequence information.

This study gives first results on the predictibility of A-minor motifs as a function of the topological context. All the classification and prediction pipelines rely on automated processes, for which we describe the underlying algorithms and parameters.

## 2. Material and methods

### 2.1. Modelling structural context of A-minor motifs by graphs

This part defines the topological structural context of a type I/II A-minor motif occurrence and explains how to represent it with graphs. Most of these notions have been defined in a previous paper [28]. Here, we summarize them and invite the reader to refer to that paper for technical details. In this article, a type I/II A-minor motif occurrence will be called an A-minor motif occurrence.

#### 2.1.1. RNA graph

We define an RNA graph as a connected directed graph $G=(V,A_{c},A_{h})$, with two sets of directed edges $A_{c}$ and $A_{h}$, also called arcs. This kind of graph representation has often been used [21–23]. Vertices of V correspond to nucleotides. The arcs of $A_{c}$ correspond to the covalent bonds of the primary sequence oriented in the 5’ → 3’ direction. And each set of hydrogen bonds between nucleotides, that we will call canonical or non-canonical interaction in the rest of this article, is represented by two arcs in $A_{h}$, in both directions. Each arc in $A_{h}$ belongs to a family corresponding to the pairing family of the interaction, according to the Leontis–Westhof nomenclature [29], in order of its direction. For example, an interaction *trans* Sugar/Hoogsteen between two nucleotides $n_{1}$ and $n_{2}$ will be represented by an arc of $A_{h}$ with the family tSH from $n_{1}$ to $n_{2}$,and by an arc of $A_{h}$ with the family tHS from $n_{2}$ to $n_{1}$. In particular, a canonical interaction is represented by two arcs with a special family (CAN), to differentiate it from the *cis* Watson-Crick/Watson-Crick family that can be non-canonical. Note that a *cis* Watson-Crick/Watson-Crick interaction between a G and a U (Wobble interaction) will be represented as two arcs of type CAN, because we consider it as part of the secondary structure even though it is not a canonical interaction.

#### Branches and k-extensions

2.1.2.

**Figure 1.**
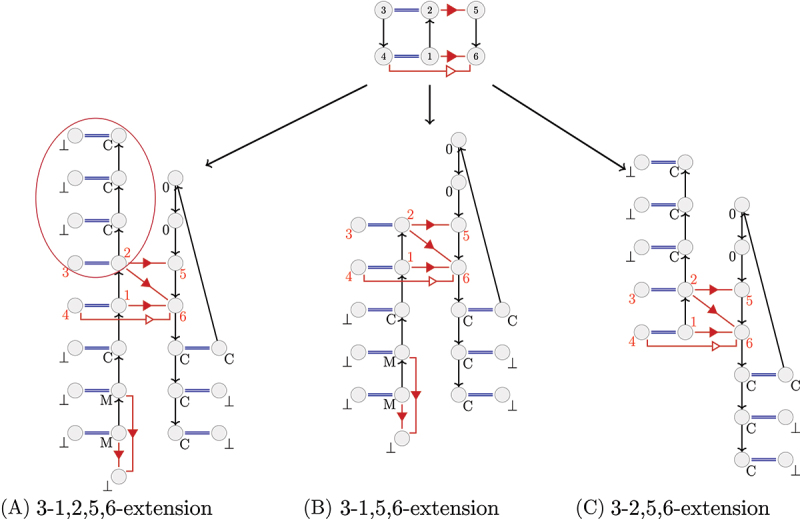
3-extensions of an A-minor motif occurrence of type I/II. In (A), the 3-extension from the nucleotides numbered 1,2,5 and 6. In (B), the 3-extension from nucleotides 1,5,6. In (C), the 3-extension from nucleotides 2,5,6. The covalent interactions are in black, the canonical interactions are represented by a double line and the non canonical interactions are represented by the corresponding symbol in the Leontis–Westhof nomenclature [29]. The long range interactions are in red and the short range interactions (belonging to a secondary structure) are in blue. Every vertex is also annotated by its label. An example of branch of length 3 from the nucleotide 2 is circled in (A).

An A-minor motif occurrence is represented by a particular RNA subgraph with six vertices numbered from 1 to 6 (see Fig. 1, top).

**Definition 2.1**. We call *branch of length* k from a vertex u of an A-minor motif occurrence, the subgraph induced by the following vertices:

• the vertex u.

• the set $V_{k}$ of vertices that are at a distance at most k from u, on a path which is composed only of arcs in $A_{c}$, whose starts or ends in u, and which does not cross any other vertex of the motif. Additionally, each of these vertices has a label according to the families of its incident directed edges in $A_{h}$: label 0 if it is unpaired, label C if involved only in a canonical interaction, label N if involved only in non-canonical interactions, label M if involved in both types of interactions.

• the set $V_{k}^{+}$ of vertices that are linked to a vertex of $V_{k}$ by an arc in $A_{h}$. These vertices have the special label ⊥.

For example, in Fig. 1A, the circled part is a branch of length 3 from the vertex number 2.

**Definition 2.2**. Let G be a RNA graph containing at least one A-minor motif occurrence and S be a subset of vertices of a given A-minor motif occurrence in G. We define the structural context from S of order k, denoted by k-S-extension, as the labelled subgraph of G induced by the following vertices:

• the six vertices of the A-minor motif occurrence,

• the vertices which belong to the branch of length k from each vertex of S.

In the remainder of the article, we will call an A-minor motif occurrence and its structural context *an embedded A-minor motif occurrence*.

In this study, we consider several possible subsets S of vertices of the motif (see Fig. 1A,B,C). The first one includes the nucleotides of the motif being involved in a *cis* Sugar/Sugar interaction (numbers 1,2,5,6 in the Fig. 1A). We thus choose to extend the motif to only one of the two strands of the helix (the strand with the nucleotides numbered 1 and 2 in the Fig. 1). The second strand, with the nucleotides numbered 3 and 4 in the Figure, will be taken into account through the canonical interactions of the helix. This extension will then be called *k-1,2,5,6-extension*.

We also consider two other subsets S of vertices: the two vertices corresponding to the nucleotides of the loop (numbers 5 and 6 in the Fig. 1B) and the vertex corresponding to the nucleotide numbered 1 in the Fig. 1B, or the two vertices corresponding to the nucleotides of the loop and the vertex corresponding to the nucleotide numbered 2 in the Fig. 1 C. The first one will be called *k-1,5,6-extension* and the second one *k-2,5,6-extension*.

These three representations will be discussed in the Results section.

#### 2.1.3. Contracted k-extension

RNA structures are subject to sequence mutation, due to evolution. Slight local changes in structures, like a difference of one nucleotide in a loop or an helix, may not change noticeably the 3D structure of the molecule, and thus may not change its function. This is why we define a contracted representation of the structural context, allowing us to represent similar but different contexts in an almost identical way.

A contracted k-extension of an A-minor motif is then a graph derived from the k-extension of the motif, in which consecutive vertices in a same strand which have the same label (as defined in Definition 2.1) are contracted in a same new vertex. This new vertex has a weight, noted p, corresponding to the number of contracted nucleotides it represents. As we will see in section 2.3.2, this contraction allows us to consider as similar pairs of helices or pairs of loops, with a difference of one or two nucleotides.

**Figure 2.**
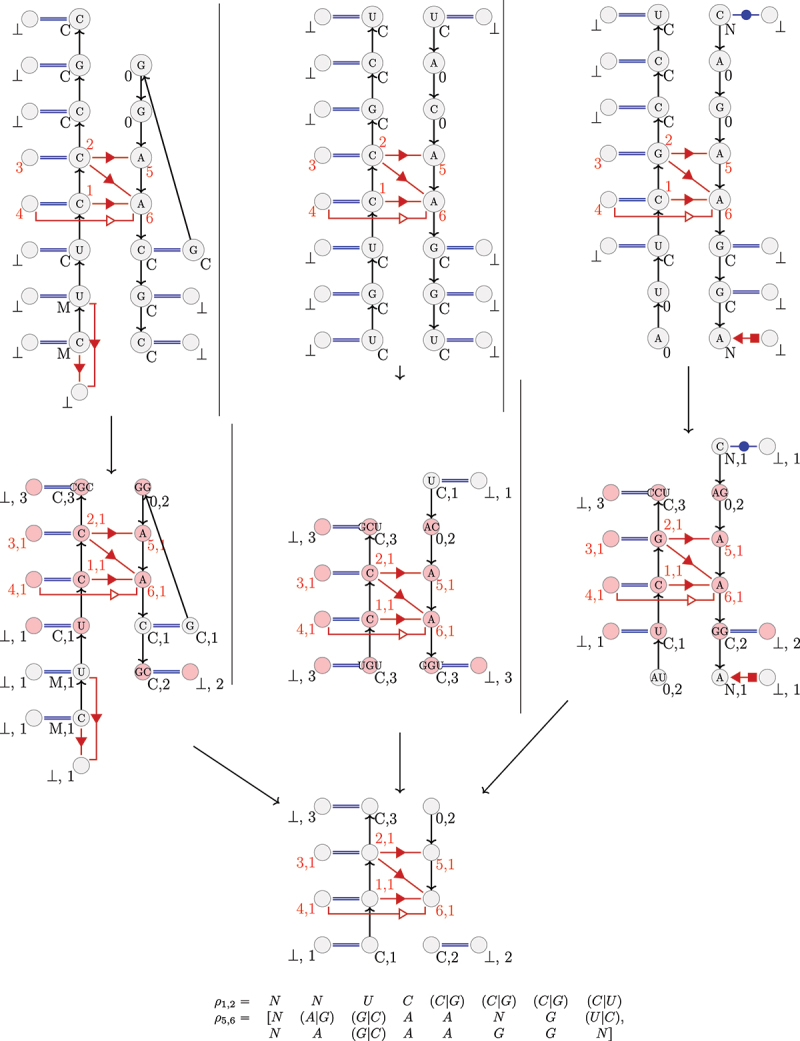
Representatives of a class of three A-minor motif occurrences. Above are represented the three uncontracted 3–1,2,5,6-extensions of the class, with the sequence in nucleotides for every vertex. In the middle, the corresponding contracted 3-extensions. The vertices in pink belong to the maximum common subgraph between the three 3-extensions . Below, are represented the maximum common subgraph (topological representative) between the three 3-extensions, and the two sets of regular expressions $\rho_{1,2}$ and $\rho_{5,6}$ (sequence representative). An ‘N’ in the regular expressions means that any type of nucleotide at this position is possible, because this position does not belong to the maximum common subgraph.

Examples of contracted k-extensions are presented in Fig. 2, second line.

### 2.2. Three-dimensional similarity between A-minor motif occurrences

**Figure 3.**
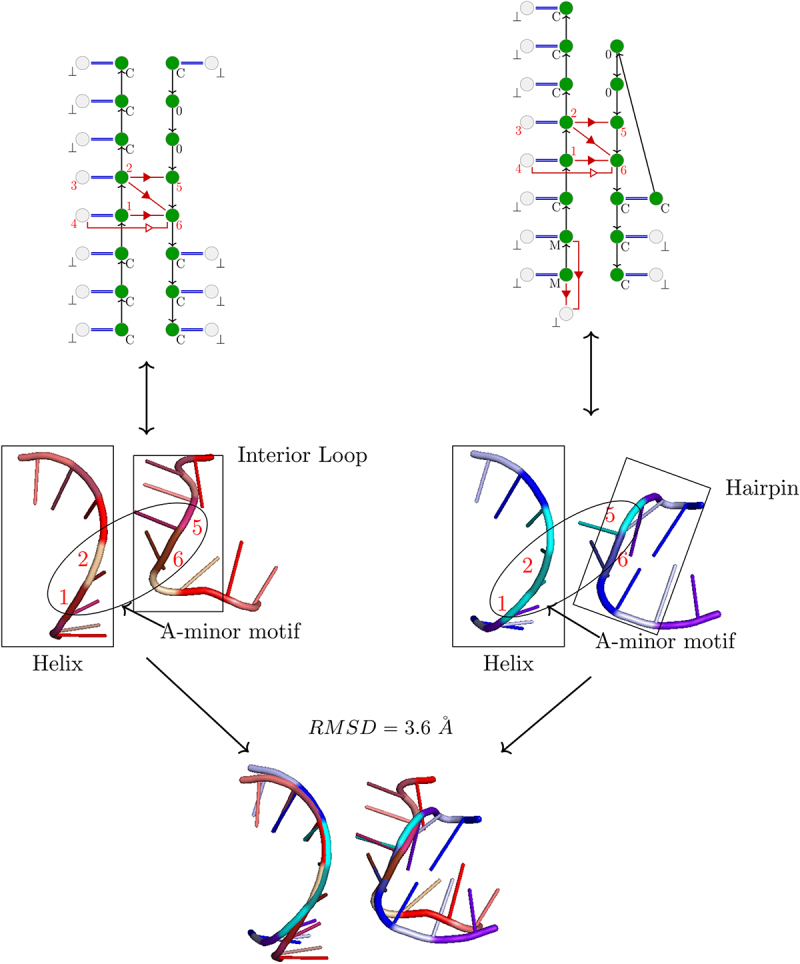
Two examples of uncontracted 3–1,2,5,6-extensions (above), the induced 3D substructures (in the middle) and their 3D alignment (below). The nucleotides in the 3D structures correspond to the vertices in green in the 3-extensions. The RMSD between the two 3D substructures is 3.6 Å, which corresponds here to 3D structures with poor 3D similarity. Only the part of helix on the top left is well aligned. The right side is particularly poorly aligned because it is an interior loop in the first case, and an hairpin in the second. In the 3D structures, only backbones and nucleotides N1 of purines and N3 of pyrimidines are represented, and the nucleotides of the A-minor motif are indicated by their number (1,2,5,6). We will use the same 3D representation in the same orientation, in the next figures.

We compare A-minor motif occurrences according to their structural context of order k, by using a measure of 3D similarity between structural contexts. For a given A-minor motif occurrence, we consider the 3D substructure induced by the corresponding k-S-extension, without the vertices from the set $V_{k}^{+}$ (see Definition 2.1). We call it a *local 3D structure*. Fig. 3 shows examples of local 3D structures of two given A-minor motif occurrences.

For comparing two such structures, each nucleotide is represented by its carbon 3’ to overcome the problem of sequence differences [30,38]. For a pair of local 3D structures, we first align two by two the six nucleotides of the A-minor motif by minimizing the RMSD [31]. Then we keep this alignment and calculate the RMSD of the two local 3D structures of the motif occurrences. An example of comparison is presented in Fig. 3.

### 2.3. Classification of A-minor motif occurrences and definitions of representatives

#### 2.3.1. Classification of A-minor motif occurrences

In order to find structural features common to several structural contexts of A-minor motif occurrences, we classify A-minor motif occurrences according to RMSD between local 3D structures around the motif occurrences.

To do so, we define an undirected weighted graph, which we call similarity graph: each vertex corresponds to an embedded A-minor motif occurrence, and there is an edge between two vertices if the RMSD between the two corresponding local 3D structures is lower than this threshold. Each edge is weighted by the corresponding RMSD.

We then apply to this graph a clustering method, named OClust-R [32], that seeks to maximize cluster density and average similarity. It also allows a motif occurrence to belong to two different classes, in order to take into account the case where one motif occurrence is close to two other motif occurrences, which are, for their part, very different.

Several classifications will be presented in the results section, corresponding to several subsets S of motif vertices (see Definition 2.2), with experimentally chosen RMSD thresholds.

#### 2.3.2. Representatives of classes

***Topological representative.*** Each class will be represented by a unique graph, that we call the *topological representative* of the class. The representative of a class is the maximum common subgraph of every contracted k-extension of this class.

We first define the maximum common subgraph between two contracted k-extensions. This common subgraph must obviously contain the vertices and edges of the A-minor motif. Furthermore, all pairs of equivalent vertices must have the same label (0, C, N, M or ⊥) and belong to the same branch (see Definition 2.1), and all pairs of equivalent edges must belong to the same pairing family. In particular, vertices with the same label but with different weights can be equivalent. This allows us to consider as similar pairs of helices or pairs of loops, with a difference of one or two nucleotides. Note, however, that in a k-extension, the weight of the vertices is between 1 and k. Therefore, for a lower value of k, as will be the case in our study (see results section), the difference in weight for two equivalent vertices remains low as well.

This common subgraph must maximize the number of edges of $A_{h}$, weighted by the weight of the incident vertices. Only edges of $A_{h}$ are taken into account because we are interested in the structural context composed of canonical and non-canonical interactions.

To find this maximum common subgraph between two contracted k-extensions, we rely on the resolution of the Maximum Common Edge Subgraph (MCES) problem [33], that aims to find a subgraph, common to any two graphs G and H, maximizing the number of edges. We adapted an exact method based on the MCES problem resolution developed in [34], which is initially used to find similarities between small molecules. The problem MCES has been proven to be NP-hard for arbitrary graphs [33], and then this exact method has an exponential complexity. However, in practice, the size of our k-extensions is small enough to keep the computation time reasonable (see resultats section).

We then define the maximum common subgraph for a class of size n, in the same way as the maximum common subgraph for two graphs, but for n graphs. To do so, we search for all the cliques of size n in a particular graph we construct from the contracted k-extensions of the class. The problem of finding all the cliques of size n is also NP-hard. However, the size of the graphs and the degrees of the vertices make possible to compute the search in reasonable time (see Results section).

The larger the size of the common subgraph, the more similar the contracted k-extensions of the class will be. An example of the maximum common subgraph between 3 k-extensions is shown in Fig. 2 (bottom).

##### 2.3.2.1. Sequence representative

We also want to characterize our classes with sequences. We construct two sets of regular expressions [35] for each class, one for each extended strand of the motif (loop and helix). They correspond to the union of all sequences of size 2(k+1) of embedded A-minor motif occurrences in the class, induced by the maximum common subgraph of the class. It means that, for each position in the sequences, the type of nucleotides (A,C,G,U) is taken into account only when the nucleotide belongs to the maximum common subgraph. Otherwise, every letter (A,C,G,U) is authorized. The regular expressions are given in the 5’→ 3’ order. An example of a pair of regular expressions is shown in Fig. 2 for a class of 3–1,2,5,6-extensions.

### 2.4. Search for class predictibility

The question is to determine how much knowledge of the context around an A-minor motif can help to infer its presence (and position). For answering this question, we define four kinds of contexts of A-minor motif. Then for each class and each kind of context, we search for occurrences of the context in the BGSU 2020 dataset (see section 3.1), and we look at whether an A-minor motif is present or not within the context.

**Figure 4.**
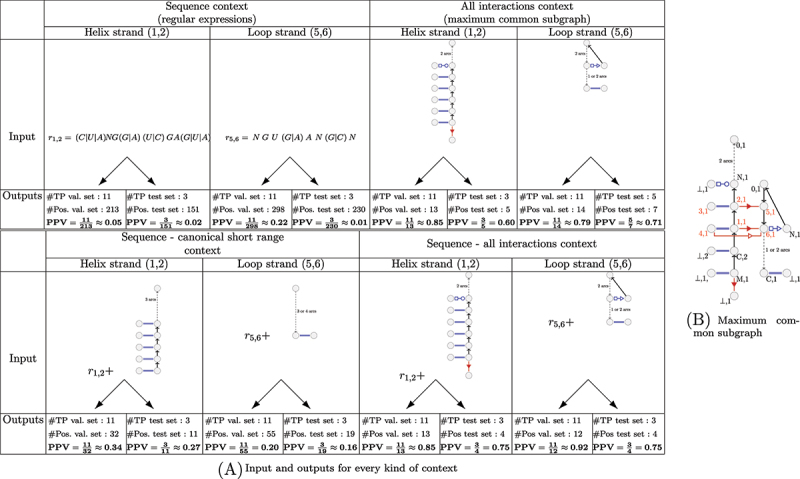
Examples of how PPV is computed for a given class, for every context (sequence, all-interactions, sequence-canonical-short-range, sequence-all-interactions). The maximum common subgraph of the class is presented on the right. The input is indicated for every context (regular expressions and/or subgraphs). For the regular expressions, the letter N indicates that every letter is authorized. For the subgraphs, the non-canonical edges in blue (resp. red) are short range (resp. long range). Every input is searched in the sequences and/or RNA graphs contained in one of the two datasets (validation (val.) or test). The outputs indicate the number of occurrences of one strand of an A-minor motif (True positives (TP)), and the total number of occurrences (Positives (Pos.)) that were found in each dataset. With these two values, the PPV can be calculated.

We define below the four kinds of contexts and the main measures we use for quantifying the ‘predictibility’ for a given class and a given context. Examples of inputs and outputs for a given class for each context are presented in Fig. 4, and a summary of the properties of each kind of contexts is given in Table 1.

**Table 1.** Summary of the four kinds of sequence and topological contexts around an A-minor motif occurrence.

Name	Properties
Sequence context	Two sets of regular expressions, one for each extended strand of the A-minor motif
All-interactions context	The topological representative, without the non canonical edges of the A-minor motif
Sequence-all-interactions context	The all-interactions context, plus the sequence context
Sequence-canonical-short-range context	The previous one, where only the short range canonical edges are kept

**Table 2.** Number of A-minor occurrences in the 2018 A-minors dataset in terms of organisms and RNA families. In the first column, the letter in brackets indicates whether the organism belongs to Bacteria, Archaea, or Eukaryota.

Family Organism	23S rRNA	25S rRNA	28S rRNA	16S rRNA	18S rRNA	Riboswitch	Intron	Ribozyme	Total
D. radiodurans (B)	18								**18**
E. coli (B)	47			21					**68**
H. marismortui (A)	76								**76**
M. jannaschii (A)	1								**1**
S. oleracea (B)	17			9					**26**
S. aureus (B)	19			5					**24**
T. thermophilus (B)	46			33					**79**
S. cerevisiae (E)		19			9				**28**
H. sapiens (E)			8		4				**12**
L. donovani (E)					7				**7**
T. petrophila (B)						1			**1**
T. tengcongensis (B)						10			**10**
T. thermophila (E)							1		**1**
O. iheyensis (B)							6		**6**
D. iridis (E)								1	**1**
Hepatitis delta virus								1	**1**
Unspecified	2					8		5	**15**
**Total**	**226**	**19**	**8**	**68**	**20**	**19**	**7**	**7**	**374**

**Table 3.** Number of A-minor occurrences of the BGSU 2020 dataset in terms of organisms and RNA families. In the first column, the letter in brackets indicates whether the organisms belongs to Bacteria, Archaea or Eukaryota.

Family Organism	12S rRNA	28S rRNA	23S rRNA	16S rRNA	Alpha rRNA	Beta rRNA	Gamma rRNA	tRNA	RNA antitoxin	Ribozyme	Riboswitch	Others	Total
S. scrofa (E)	6												**6**
O. cuniculus (E)		8											**8**
A.baumannii (B)			12	7									**19**
P. aeruginosa (B)			13	6									**19**
T. celer (A)				6									**6**
H. sapiens (E)	1												**1**
T. cruzi (E)					4	1							**5**
L. donovani (E)					4	4	1						**9**
T. thermophilus (B)								1				1	**2**
E. coli (B)								2				2	**4**
S. enterica (B)								1					**1**
Hepatovirus A												1	**1**
C. Pelagibacter (B)											1		**1**
Geobacter (B)											1		**1**
Unspecified									1	2	1		**4**
**Total**	**7**	**8**	**25**	**19**	**8**	**5**	**1**	**4**	**1**	**2**	**3**	**4**	**87**

#### 2.4.1. Sequence context.

The *sequence context* of a given class is defined by its sequence representative (see section 2.3.2). For every class, we search for its two sets of regular expressions in every sequence in the BGSU 2020 dataset. So we get two sets of occurrences: the occurrences of the strand of the A-minor motif involving the nucleotides 1 and 2 (helix strand), and the occurrences of the strand of the A-minor motif involving the nucleotides 5 and 6 (loop strand).

To quantify the predictibility of the class, we are then interested in the proportion of actual A-minor motif occurrences among the results. This measure is generally known as the Positive Predictive Value (PPV) or Precision [36]. It calculates the number of true positives out of the total number of positives. In our case, for a given class C, we will calculate a PPV for the loop strand and a PPV for the helix strand, as we have two sets of occurrences.

(1) $$PPV(C,loop)=\frac{\begin{array}{c} \#loop\;strand\;occurrences\;involved\;in\\ \;an\;A-minor\;occurrence\;of\;C \end{array}}{\#loop\;strand\;occurrences}$$

(2) $$PPV(C,helix)=\frac{\begin{array}{c} \#helix\;strand\;occurrences\;involved\\ \;in\;an\;A-minor\;occurrence\;of\;C \end{array}}{\#helix\;strand\;occurrences\;}$$

In both calculations, the number of true positives in the numerator corresponds to the number of occurrences of the loop strand (equation (1)) or of the helix strand (equation (2)), that are involved in an A-minor motif occurrence of the class. The number of false positives is thus the number of occurrences of the loop strand (resp. helix strand) that are not involved in an A-minor motif occurrence of the class. We thus can note that, for a given class, if the regular expressions associated with the loop strand (resp. helix strand) matches the loop strand (resp. helix strand) of an A-minor motif occurrence of another class, this occurrence will be counted as false positive.

Note also that, for a pair of regular expressions that represents a given class, we potentially find several loop strands and several helix strands in an RNA sequence. We cannot decide which ones of them are corresponding, without having more information on the structure.

We also use the True Positive Rate, that is the number of actual A-minor occurrences that are found (True Positives), divided by the total number of A-minor occurrences that are present in the dataset (True Positives plus False Negatives):

(3) $$TPR=\frac{\#actual\;A-minor\;occurrences\;found}{\#actual\;A-minor\;occurrences}$$

We consider here that an A-minor motif occurrence is found when both its strands are found, using the sequence representatives of the loop strand and the helix strand of the same class.

#### 2.4.2. All-interactions context.

The *all-interactions context* of a class of A-minor motif occurrences is defined as its topological representative (see section 2.3.2), where all the non-canonical edges of the A-minor motif have been removed. In most cases, removing these edges from the topological representative disconnects it, giving two subgraphs, one for the helix strand and one for the loop strand. Thus, we can use the PPVs defined above, for the loop strand and for the helix strand. In the few cases where interactions between the strands exist other than the A-minor motif, we have a single set of occurrences, corresponding to the occurrences of the unique graph. We thus calculate the number of true positives as the number of occurrences corresponding to both strands of an A-minor motif occurrence of the class, and the number of positives as the total number of occurrences. For convenience in the results, we consider for these classes the two PPVs with the same value.

#### 2.4.3. Combining sequence and topology.

We also consider two other kinds of contexts, that mix sequence features and topological features. The first one, that we call *sequence-all-interactions context*, is composed of the all-interactions context, plus the sequence context. And the second one, that we call *sequence-canonical-short-range context* is composed of the sequence context, plus the all-interactions context, by keeping only the short range canonical edges. For these two mixed contexts, we have in general two sets of occurrences, one per strand, as it is the case for the two other previously defined contexts.

For the sequence-canonical-short-range context, we thus search simultaneously for the topological context of a given class, reduced to its short range canonical edges, and its regular expressions. For the helix strand, this context contains at least part of an helix, but can also contain a part of a loop. Similarly, the topological context of the loop strand of a given class can include a part of helix (see Fig. 4, sequence-canonical-short-range context).

## 3. Data

### 3.1. Datasets

We use two different types of datasets. The *2018 A-minors dataset* is composed of 374 embedded A-minor motif occurrences. And the *BGSU 2020 dataset* is composed of 1582 non-redundant RNA structures of various sizes from the PDB. The content of these datasets is detailed in Supplemental material.

#### 3.1.1. 2018 A-minors dataset

The 2018 A-minors dataset is composed of 374 intramolecular non-redundant occurrences of type I/II A-minor motif occurrences from PDB structures with a resolution of 3 Å or better. These occurrences are taken from the database CaRNAval [23] (November 2018) and the structural contexts of these occurrences are taken from the PDB and annotated with the FR3D programme [18]. The vast majority of the A-minor motif occurrences are originated from ribosomes (91%), however the dataset also contains occurrences from ribozymes, riboswitches and introns (Table 2). These molecules are found in 16 different organisms, including 8 bacteria, 2 archaea, 5 eukaryotes and 1 virus, and there are 15 occurrences for which the organisms are unspecified.

Occurrences of this dataset are listed in Supplementary material (S1.xls).

#### 3.1.2. BGSU 2020 dataset

This dataset contains the representatives of equivalence classes from the BGSU RNA group (release 3.136, retrieved in June 2020) [37], with a resolution of 3 Å or better. It is composed of 1582 non-redundant RNA structures of various sizes from the PDB.

For every RNA molecule of this dataset, we extracted its RNA graph (see section 2.1) by using the FR3D annotation program [18].

This dataset can be divided in two parts. The first part, that we call the validation set is composed of the molecules from where the 374 A-minor motif occurrences of CaRNAval have been extracted (see Table 2). It is composed of 136 structures of molecules.

The second part, called the test set, is composed of all the other PDB structures. It is composed of 1446 structures of molecules and contains 87 A-minor motif occurrences (see Table 3 and Table 4). These 87 A-minors motif occurrences are found in 29 PDB structures. Note that none of the molecules of the test set has been used for building the 2018 A-minors dataset. Note also that most of the molecules of this dataset do not contain any A-minor motif.

**Table 4.** Number of PDB structures by RNA family in the BGSU 2020 dataset (without the 29 structures with A-minor occurrences)

RNA family	Number of PDB structures
tRNA	62
Riboswitch	37
5S rRNA	19
Ribozyme	18
snRNA	14
crRNA	12
mRNA	8
virus RNA	8
sgRNA	7
16S rRNA	6
23S rRNA	6
SRP	6
5.8S rRNA	3
gamma rRNA	1
epsilon rRNA	2
zeta rRNA	2
RNA antitoxin	2
intron	1
delta rRNA	1
28S rRNA	1
Others (sequence size superior to 50)	84
Others (sequence size inferior to 50)	1117
**Total**	**1417**

These two datasets are described in Supplementary material (S1.xls).

### 3.2. Homology groups

To determine how our classifications of A-minor motif occurrences behave with respect to homology, we detect occurrences that could be considered as homologous. We are interested in homologous occurrences, firstly in order to test the relevance of our approach (see Resultats section), and secondly in order to distinguish similar structural contexts due to homology or due to convergence. A set of homologous occurrences will be called an homology group.

#### 3.2.1. Detection of homologous A-minor motif occurrences using Gutell alignments

To detect them, we first use the sequence-structure alignments of Gutell [39] (retrieved in April 2021). A-minor motif occurrences from homologous molecules that are aligned in these alignments will be considered as homologous. In these alignments, 75% of the A-minor motif occurrences of the 2018 A-minors dataset are found.

#### 3.2.2. Detection of homologous A-minor motif occurrences using a dedicated method

For the 25% of occurrences that are not present in Gutell’s alignments, we developed a specific method. We search for pairwise sequence similarity and structure similarity among the A-minor motif occurrences from homologous molecules. Thus, we perform a global alignment of the sequences of 30 nucleotides on both directions around each strand of the motif, for each pair of occurrences of the 2018 A-minors dataset. To do so, we apply the Needleman-Wunsch algorithm implemented in the EMBOSS Needle tool from EMBL-EBI [40], with standard parameters (gap opening penalty of 10, and gap extending penalty of 0.5). We thus obtain three alignments for each pair of A-minor motif occurrences, one for each strand of the motif. We also compare the local 3D structures by using RMSD as described in section 2.2.

We then establish two thresholds to discriminate homologous occurrences: one for the alignment scores and one for the RMSD. Two occurrences are considered homologous if both alignment scores are above the corresponding threshold, and RMSD is under the corresponding threshold.

The thresholds we choose are 2Å for the RMSD, and 50 for the alignment score. We test the validity of the method by checking if the homologous occurrences, found among the A-minor motif occurrences in the Gutell’s alignments, are also obtained with our method. This is the case for almost all pairs of occurrences: only 7 pairs of occurrences are not correctly classified with our method, on a total of more than 35000 pairs.

Next, we construct a graph where each vertex is an A-minor motif occurrence, and there is an edge between two occurrences if they are homologous. Finally, we group homologous occurrences with the single link approach: two occurrences are in the same homology group if they belong to the same connected component in the graph. In this way, we obtain 49 groups of 3 or more homologous occurrences, 27 groups of 2 occurrences, and 11 occurrences not homologous to any other occurrence, in the 2018 A-minors dataset.

## 4. Results

This section presents the results obtained using the datasets we previously presented.

In a first part, several classifications of the occurrences of the 2018 A-minors dataset, based on 3D similarities (see section 2.2) will be presented. For every classification, we fixed an order of extension k equal to 3. This value of k was chosen experimentally, among a range between 1 and 10. It allows us to optimize the consistency between 3D similarity (through RMSD, see section 2.2) and graph similarity, as it is described in [28]. By choosing a k equal to 3, we can also note that our 3D classification will easily distinguish between A-minor motif occurrences involving a tetraloop and A-minor motif occurrences involving an internal loop, because RMSD will be higher between a tetraloop and an internal loop than between two tetraloops or two internal loops.

The first classification is based on 3–1,2,5,6-extensions (see Definition 2.2). It will be called 1,2,5,6-classification. The two other classifications are based on extensions around only 3 nucleotides of the motif (3–1,5,6-extension and 3–2,5,6-extension). These classifications will be, respectively, called 1,5,6-classification and 2,5,6-classification. We will describe these three classifications and their particularities.

In a second part, we will present the results of the predictibility study, on the classes of the 1,2,5,6-classification, by using the four kinds of context defined in section 2.4.

The source code of this study implemented in Python3 is freely available at https://cbe.uqam.ca/aminor_analysis/, along with a jupyter notebook to test some features. Note that the search for topological representatives for all the classes takes around 5 hours on a PC (Intel Core i5-7440HQ 4 × 2.80GHzCPU), and the search for occurrences of representatives in the two datasets (validation and set) takes around a day on the same PC.

### 4.1. Classifications based on 3D similarities

For every classification, we present in this section, details about the content of each class are available in Supplemental material (S1.xls).

#### 4.1.1. Classification on 4 branches: 1,2,5,6-classification

##### 4.1.1.2. Description of the classification

With an order k equal to 3 and an extension around 4 nucleotides of the motif, the local 3D structures around the A-minor motif occurrences are composed of 18 nucleotides. To cluster these A-minor motif occurrences according to the similarity of their local 3D structures, with the method described in 2.2, we choose a threshold on RMSD of 2.5Å. This threshold was chosen experimentally to obtain relevant 3D alignments (see examples in Fig. 6).

The number of classes per size is indicated in Table 5, in comparison with the number of classes of the same size in the two other classifications.

**Table 5.** Number of classes by size for each classification (RMSD threshold of 2.5Å).

Class sizeClassification	*1*	*2*	*3*	*4*	*5*	*6*	*7*	*8*	*9*	*10-19*	*20-29*	*30-39*	*40-49*	*50-59*	*Total*
*1,2,5,6-classification*	19	10	13	10	1	3	2	5	2	13	1	0	0	0	**79**
*1,5,6-classification*	19	8	14	8	1	2	1	4	2	10	0	1	0	1	**71**
*2,5,6-classification*	17	7	12	12	1	1	2	4	2	9	3	0	0	1	**71**

The distribution of non-ribosomal A-minor occurrences in this classification is presented in Table 6. We see in this table that 4 classes are composed of non-ribosomal A-minor motif occurrences only, and 5 classes possess ribosomal and non-ribosomal occurrences.

**Table 6.** Proportion of A-minor occurrences found in non-ribosomes for every class of the 1,2,5,6-classification where at least one A-minor motif occurrence is found in non-ribosomes. The proportion x/y indicates that there are x occurrences of non ribosomal occurrences out of y occurrences in the class.

Class id	Non ribosome proportion	RNA families in the class
41	3/3	Intron
43	1/9	23S rRNA, 25S rRNA, Ribozyme
45	13/13	Riboswitch
49	2/6	16S rRNA, Intron
50	1/17	23S rRNA, 25S rRNA, 28S rRNA, 18SrRNA, 16S rRNA, Intron
54	3/3	Intron, Ribozyme
56	2/26	23S rRNA, 25S rRNA, 28S rRNA, Riboswitch
57	4/4	Riboswitch
59	1/15	23S rRNA, 25S rRNA, Intron

##### 4.1.1.3. Consistency of the classification with homologous occurrences

**Figure 5.**
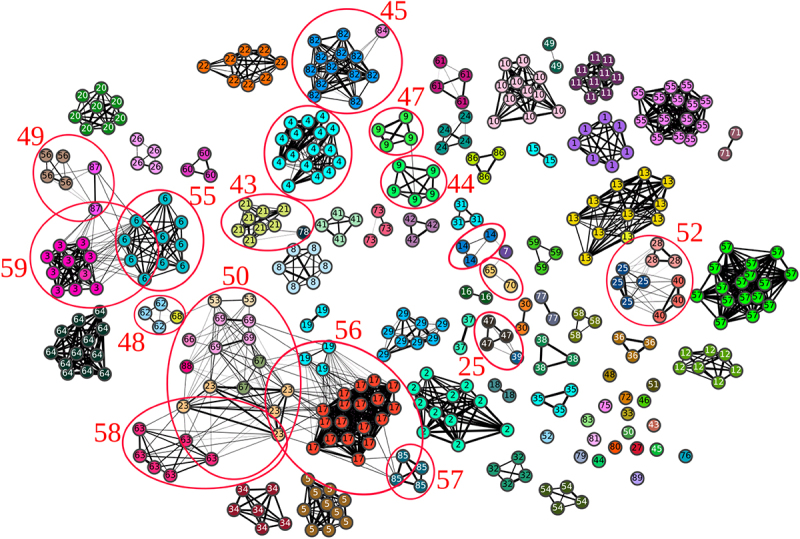
Similarity graph representing the 2018 A-minors dataset and 1,2,5,6-classification of this dataset. Each vertex is an embedded A-minor motif occurrence and there is an edge between two vertices if the RMSD is equal to 2.5Å or better. The thicker the edges, the lower the RMSD. Homologous occurrences have the same colour and the same number. Examples of classes are circled in red (the other classes correspond to connected components of the graph) .

This 1,2,5,6-classification is consistent with homology. Two homologous occurrences are generally grouped together, and most of the classes contain only homologous occurrences. More precisely, according to our RMSD threshold, 1305 pairs of occurrences are homologous and share similar local 3D substructures, whereas 242 non-homologous pairs share similar 3D substructures and 50 homologous pairs do not share similar 3D substructures (see Fig. 5).

In the following paragraph, we will detail some examples of 3D classes composed of non-homologous occurrences and examples of homologous occurrences not grouped in the same 3D class.

##### 4.1.1.4. Differences between homology groups and 3D similarity classes

**Figure 6.**
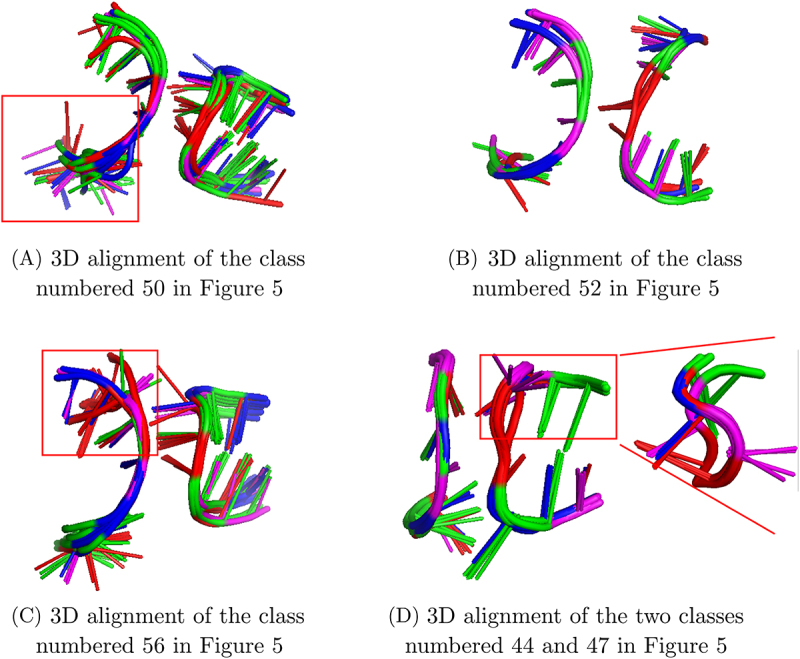
3D alignments of examples of classes from the 1,2,5,6-classification. These classes are composed of non homologous occurrences (A), (B), (C) or composed of homologous occurrences that are split into two classes (D). The structures are coloured by nucleotide type (A:red, C:blue, G:green, U:magenta). The same 3D representation as in the Fig. 3 is used. In (A) and (C)., the less well aligned part of the 3D structures are bordered in red. In (D), the less well aligned part is bordered in red and displayed in a different orientation.

As it can be seen in Fig. 5, 11 classes are composed of non-homologous occurrences. For example, the class numbered 50 is constituted of occurrences from 6 homology groups, found in both subunits of ribosomes or in introns, from eukaryotic or bacterial organisms. The class numbered 52 is constituted of occurrences from 3 homology groups all found in large subunits of ribosome, in organisms from Bacteria and Archaea. The class numbered 56 is composed of occurrences from 4 homology groups from large subunits of ribosome or from riboswitches. The organisms in the class 56 belong to the three domains of life. The 3D alignments of these three classes are presented in Fig. 6A,B,C.

On the other hand, the classes 44 and 47 are composed of homologous occurrences. One of these classes (class 44) is actually composed of occurrences from Bacteria and the other (class 47) is composed of occurrences from Archaea and Eukaryota. The 3D difference between the structural contexts of these occurrences can thus be explained by evolutive drift. The 3D alignment of these two classes is presented in Fig. 6D. The major difference in this alignment is the part at the top right on the Figure, framed in red, where the nucleotides from the class 47 and the nucleotides from the class 44 are not aligned at all.

However, it can be noticed that, in some cases, the classes of non-homologous occurrences do not group all the occurrences of one given homology group (example of classes 56, 58 or 59). Furthermore, the 3D alignments presented in Fig. 6 show that one branch of the extension is often less well aligned than the others. This less well-aligned branch often belongs to the helix strand (Fig. 6A and 6C). Some parts of the 3D contexts of A-minor motif occurrences, in particular the loop strand, thus seem to be more conserved than the others. This structural asymmetry may suggest that one part of the 3D context is more important than the other for the motif formation. This is what we will investigate in the following, by calculating other classifications, considering only three branches around the A-minor motif. The results are presented in the next part.

#### 4.1.2. Classifications on 3 branches

This part describes the 3D classifications obtained from extensions around three nucleotides of the motif: the two nucleotides involved in the loop (numbered 5 and 6) and one of the two nucleotides involved in the helix and in a *cis* Sugar/Sugar interaction (numbered 1 or 2). The local 3D structures induced by these types of extension are then composed of 15 nucleotides.

We choose the same RMSD threshold of 2.5Å as for the classification on 4 branches. Smaller classes composed of non-homologous occurrences, obtained from the same 3 branches, and with a RMSD threshold of 2 Å, are described in Supplementary material (S2).

The number of classes per size is shown in Table 5 for both classifications, along with the values for the classification on 4 branches.

**Figure 7.**
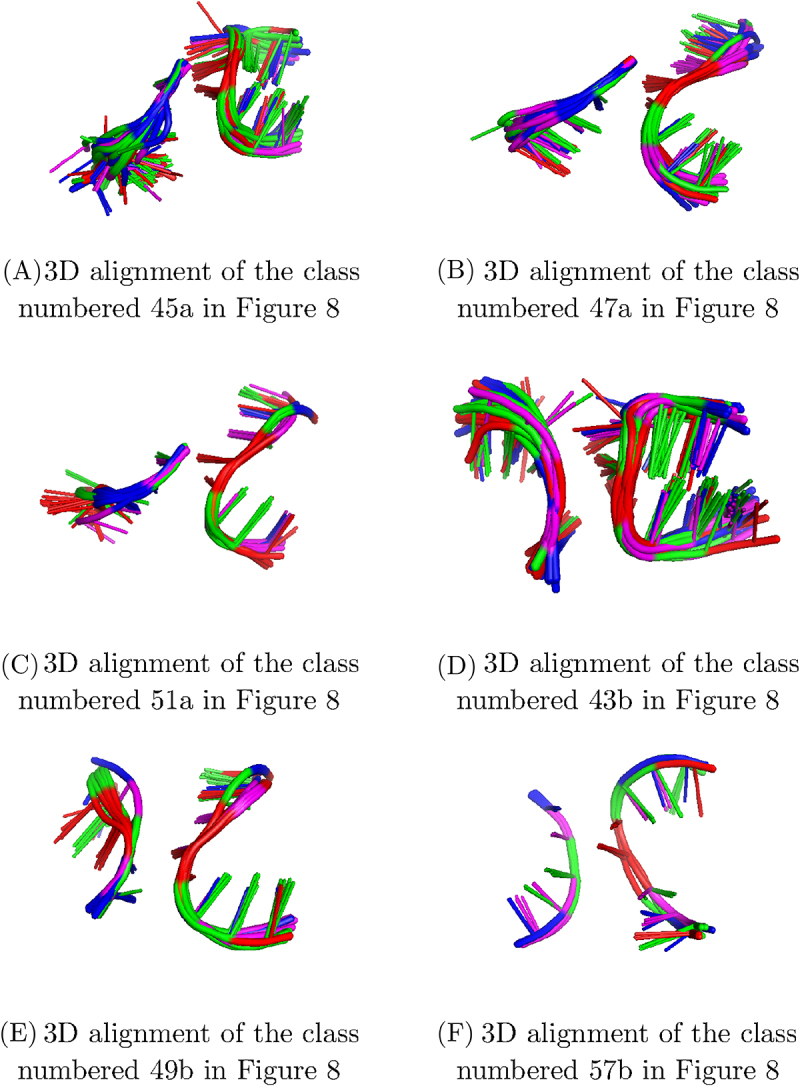
3D alignments of local 3D structures of A-minor motif occurrences from classes in the 1,5,6-classification ((A), (B), (C)) or in the 2,5,6-classification ((D), (E), (F)) with non homologous occurrences. The 3D structures are coloured by nucleotide type (A: red, C: blue, G: green, U: magenta), with the same 3D representation as in Fig. 3. For each 3D structure, only the 3 branches considered in the classification are represented. The branch 2 is missing in (A), (B), (C) and the branch 1 is missing in (D), (E) and (F).

These two classifications, called 1,5,6-classification and 2,5,6-classification, group pairs of homologous occurrences together, as the classification on 4 branches. However, several new classes of non-homologous occurrences appear. We will be particularly interested in the classes, from these two classifications, with occurrences from at least two homology groups, and in which there are more than one occurrence by homology group if the class is composed of only two homology groups. These classes are presented in the next paragraphs and in Fig. 7.

In order to distinguish the class numbers of the different classifications, we will use the suffix ‘a’ to refer to the 1,5,6-classification and the suffix ‘b’ to refer to the 2,5,6-classification. For example, class number 50 of the 1,5,6-classification will be called 50a and class number 50 of the 2,5,6-classification will be called 50b.

**Figure 8.**
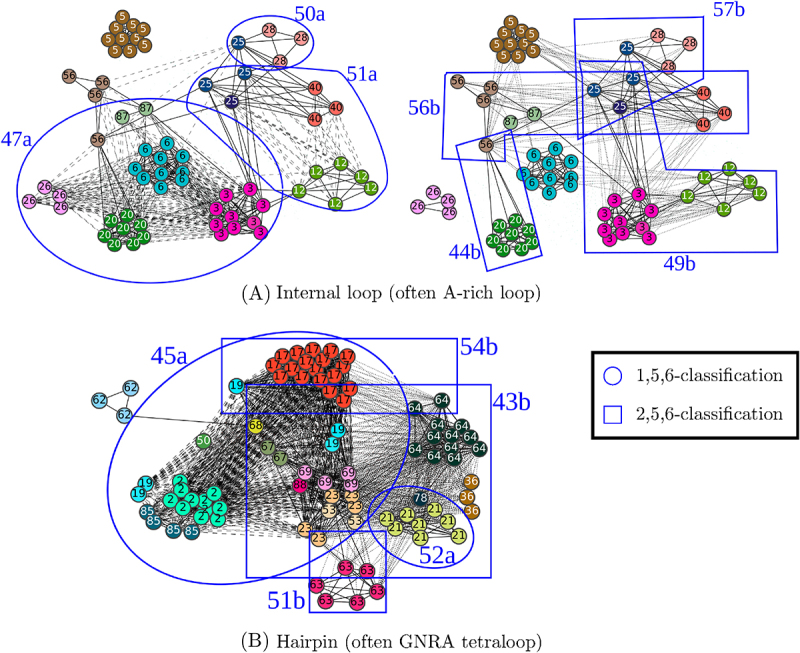
Similarity graphs representing the classes from the 1,5,6-classification (circled in blue and annotated with ‘a’) and from the 2,5,6-classification (framed in blue and annotated with ‘b’),with non homologous occurrences. The vertices correspond to the embedded A-minor motif occurrences. Homologous occurrences have the same colour and the same number. There is an edge, represented by a solid line, between two vertices if the RMSD (in both classifications) between the corresponding local 3D structures is equal to 2.5 Å, between two vertices if the RMSD between the corresponding local 3D structures in the 1,5,6-classification only (resp. 2,5,6-classification only) is equal to 2.5 Å or better. There is an edge, represented by a dotted line (resp. a dashed line), between two vertices if the RMSD between the corresponding local 3D structures in the 1,5,6-classification only (resp. 2,5,6-classification only) is equal to 2.5 Å or better. For each connected component, the type of loop in the A-minor motif occurrences is indicated.

**1,5,6-Classification** The 1,5,6-classification is composed of 71 classes (see Table 5 for details) and comprises 3 classes that fit the criterion of non homology given above (classes 47a, 51a and 45a circled in blue in Fig. 8) . The 3D alignments of these three classes are presented in Fig. 8.

The first one (numbered 47a in the Fig. 8) is composed of 34 occurrences from 6 different homology groups. The occurrences are found in ribosomes or in introns, from Bacteria, Archaea and Eukaryota. Every occurrence of this class involves an internal loop, and in half of the cases, this internal loop is a loop including a tSH and a tWH interactions and the nucleotides 5 and 6 are adenines, so it is called A-rich loop. This class especially contains the occurrences of the classes 55 and 59 of the 1,2,5,6-classification on 4 branches (see Fig. 5).

The second class (numbered 51a in the Fig. 8) contains 12 occurrences from 3 different homology groups. The occurrences of this class are all found in large subunits of ribosome, from Bacteria, Archaea and Eukaryota. Every occurrence of this class involves an internal loop, and, in particular, 5 of them involve an A-rich loop.

The third class (numbered 45a in the Fig. 8) contains 54 A-minor motif occurrences from 11 homology groups. The occurrences of this class are found in various molecules, such as both subunits of ribosomes, riboswitches and introns, in organisms belonging to Bacteria, Archaea and Eukaryota. All of the occurrences of this class involve an hairpin, and in most cases, this hairpin is a GNRA tetraloop [41]. However, note that other classes also involve motif occurrences with a GNRA loop. In comparison with the 1,2,5,6-classification, this class groups 8 classes of the 1,2,5,6-classification, in particular the classes of non-homologous occurrences, numbered 50 and 56 (see Fig. 5) .

**2,5,6-Classification** The 2,5,6-classification is composed of 71 classes (see Table 5 for details) and comprises 4 classes that fit the criterion of non homology (classes 49b, 56b, 57b and 43b framed in blue in Fig. 8) . The 3D alignments of these 4 classes are presented in Fig. 7.

The first one (numbered 49b in the Fig. 8) is composed of 19 occurrences from 3 different homology groups. The A-minor motif occurrences are all found in large subunits of ribosome, from Bacteria, Archaea or Eukaryota. They all involve an internal loop, that is often an A-rich loop (12 out of 19).

The second one (numbered 56b in the Fig. 8) is composed of 12 occurrences from 4 different homology groups. The occurrences of this class are found in both subunits of ribosomes or in introns, from bacterial or archaeal organisms. They all involve an internal loop which is not an A-rich loop.

The third one (numbered 57b in the Fig. 8) is composed of 7 occurrences from 2 different homology groups. The occurrences are found in large subunits of ribosome, from Bacteria or Archaea. As the class 56b presented above, the occurrences of this class all involve an internal loop, which is not an A-rich loop.

The last class that we consider in the 2,5,6-classification (numbered 43b in the Fig. 8) is composed of 48 occurrences from 15 homology groups. The occurrences are found in both subunits of ribosomes, in introns or in ribozymes, from Bacteria, Archaea or Eukaryota. All of the occurrences of this class involve an hairpin that is almost always a GNRA tetraloop (except 3), like the class 45a of the 1,5,6-classification. These two classes (43b of the 2,5,6-classification and 45a of the 1,5,6-classification) possess 20 occurrences in common, and 15 of them were already gathered in the 1,2,5,6-classification on 4 branches (class 50, Fig. 5), along with 2 other occurrences.

**Figure 9.**
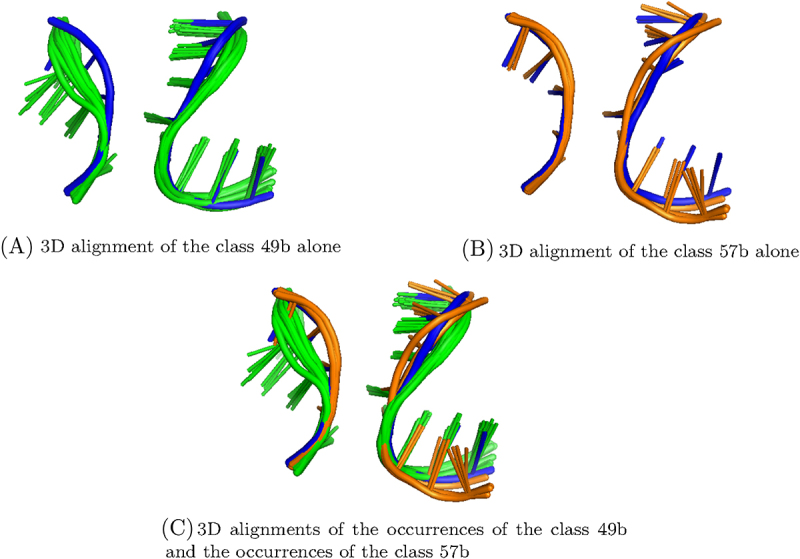
3D alignment of the classes 49b and 57b of the 2,5,6-classification (see Fig. 8). The green structures are part of the class 49b only, and the orange structures are part of the class 57b only. The blue structures belong to both class 49b and class 57b (in greyscale, it corresponds to the darkest).

It can be noted that the 2,5,6-classification contains several classes containing A-minor motif occurrences in common (see Fig. 8). For example, the classes 49b, 56b and 57b possess 3 A-minor motif occurrences in common. In Fig. 9, are presented the 3D alignments of the local 3D structures of the occurrences from classes 49b and 57b. It shows that the local 3D structures of occurrences that are in both classes (in blue) seem to share similarities with the local 3D structures of the occurrences that are only in one of the two classes (in green and orange). It then justifies that the A-minor motif occurrences that the classes have in common actually belong to both classes.

### 4.2. Towards prediction of A-minor motifs

What kind of information, contained in the structural context, can be useful to characterize and predict the presence and the position of A-minor motifs? This is the question we address in this section. In section 2.4 we defined four kinds of sequence and topological contexts for a class of embedded A-minor motif occurrences, and we defined two measures for quantifying how a given context can infer the presence of an A-minor motif occurrence in a RNA graph (see **section 2.4.1**): the PPV by strand and by class, and the TPR which we calculate for all classes together. Here we focus on the 1,2,5,6-classification, and we consider only the classes which contain at least 3 occurrences in the 2018 A-minors dataset. We show the results of predicting A-minor motifs in the BGSU 2020 dataset, given their contexts. All PPV values per class are detailed in Supplementary material (S1.xls). Remember that, as explained in section 3.1, the BGSU 2020 dataset is divided in two parts, the validation set and the test set.

#### 4.2.1. Average PPV and TPR to compare topology and sequence contexts on both strands

Table 7 shows the average PPV for the loop strand and for the helix strand over all the classes, in the validation set. And Table 8 shows the average PPV for each strand in the validation set and in the test set, for the classes having at least one occurrence of an embedded A-minor motif occurrence in the test set. We consider that an embedded A-minor motif occurrence in the test set belongs to a given class if its RMSD with at least one occurrence of the class (in the validation set) is less or equal to 2.5 Å. As we can see in Table 7 and Table 8, there is a good correlation between results in the two sets for both strands. Meanwhile, the absolute PPV values between the two sets cannot be compared directly, because the respective sizes and properties of the data are not the same. As expected, the best PPV values are obtained for the combination of sequence and all interactions, respectively, for the (complete) validation set, 0.59 for the loop strand and 0.54 for the helix strand, and, for the test set, 0.36 for the loop strand and 0.33 for the helix strand

**Table 7.** Average PPV values for each strand, for each category of context, with the validation set.

Category of context	Mean PPV validation set	
	Loop strand	Helix strand
Sequence context	0.16	0.13
All-interactions context	0.31	0.23
Sequence-canonical-short-range context	0.32	0.27
Sequence-all-interactions context	0.59	0.54

**Table 8.** Average PPV values, for each category of context, with the validation set and the test set, for the classes having at least one occurrence of an embedded A-minor motif in the test set.

Category of context	Mean PPV validation (sub)set		Mean PPV test set	
	Loop strand	Helix strand	Loop strand	Helix strand
Sequence context	0.08	0.07	0.02	0.01
All-interactions context	0.24	0.15	0.19	0.13
Sequence-canonical-short-range context	0.21	0.17	0.12	0.09
Sequence-all-interactions context	0.51	0.47	0.36	0.33

There is a drastic global augmentation of the PPV, for the sequence and canonical short range context compared to the sequence context (for the validation set, 0.16 to 0.32 on average for the loop strand, and 0.13 to 0.27 on average for the helix strand, and for the test set, 0.02 to 0.12 on average for the loop strand, and 0.01 to 0.09 for the helix strand).

Similarly, the average PPV is considerably better for the all interactions context than for the sequence context (for the validation set, 0.16 to 0.31 on average for the loop strand and 0.13 to 0.23 on average for the helix strand, and for the test set, 0.02 to 0.19 on average for the loop strand, and 0.01 to 0.13 on average for the helix strand).

On the other hand, taking both the sequence and the canonical short-range interactions gives no advantage, globally, to predict A-minor motif occurrences compared to the all-interactions context (without sequence), especially for the loop strand (average PPV of 0.31 and 0.32 for the validation set). This is even the contrary in the test set (mean PPV of 0.12 for the sequence-canonical-short-range context and 0.19 for the all-interactions context).

Moreover, the average PPV values are better for the loop strand than for the helix strand, for every context and every dataset. This is especially the case for the all-interactions context in the validation set, where the average PPV for the loop strand is 0.31 and 0.23 for the helix strand.

Table 9 shows the global True Positive Rate (TPR) on both datasets. For both datasets, the results are fairly good. Obviously, in the validation set, all the A-minor motifs belonging to a class with more than 3 A-minor motif occurrences are found, since all these classes and only them have been taken into account for this study. The missing A-minor motif occurrences in this dataset are in classes of size 2 or isolated in the classification. In the test set, more than 60% of the motif occurrences are found with the sequence and all interactions context, which is the most restrictive one.

**Table 9.** True Positive Rate (TPR) on both datasets.

Category of context	True Positive Rate validation set	True Positive Rate test set
Sequence context	368/374 ≈ 0.98	76/87 ≈ 0.87
All-interactions context	351/374 ≈ 0.94	67/87 ≈ 0.77
Sequence-canonical-short-range context	341/374 ≈ 0.91	57/87 ≈ 0.66
Sequence-all-interactions context	341/374 ≈ 0.91	53/87 ≈ 0.61

#### 4.2.2. Classes with different profiles according to the predictibility contexts

**Figure 10.**
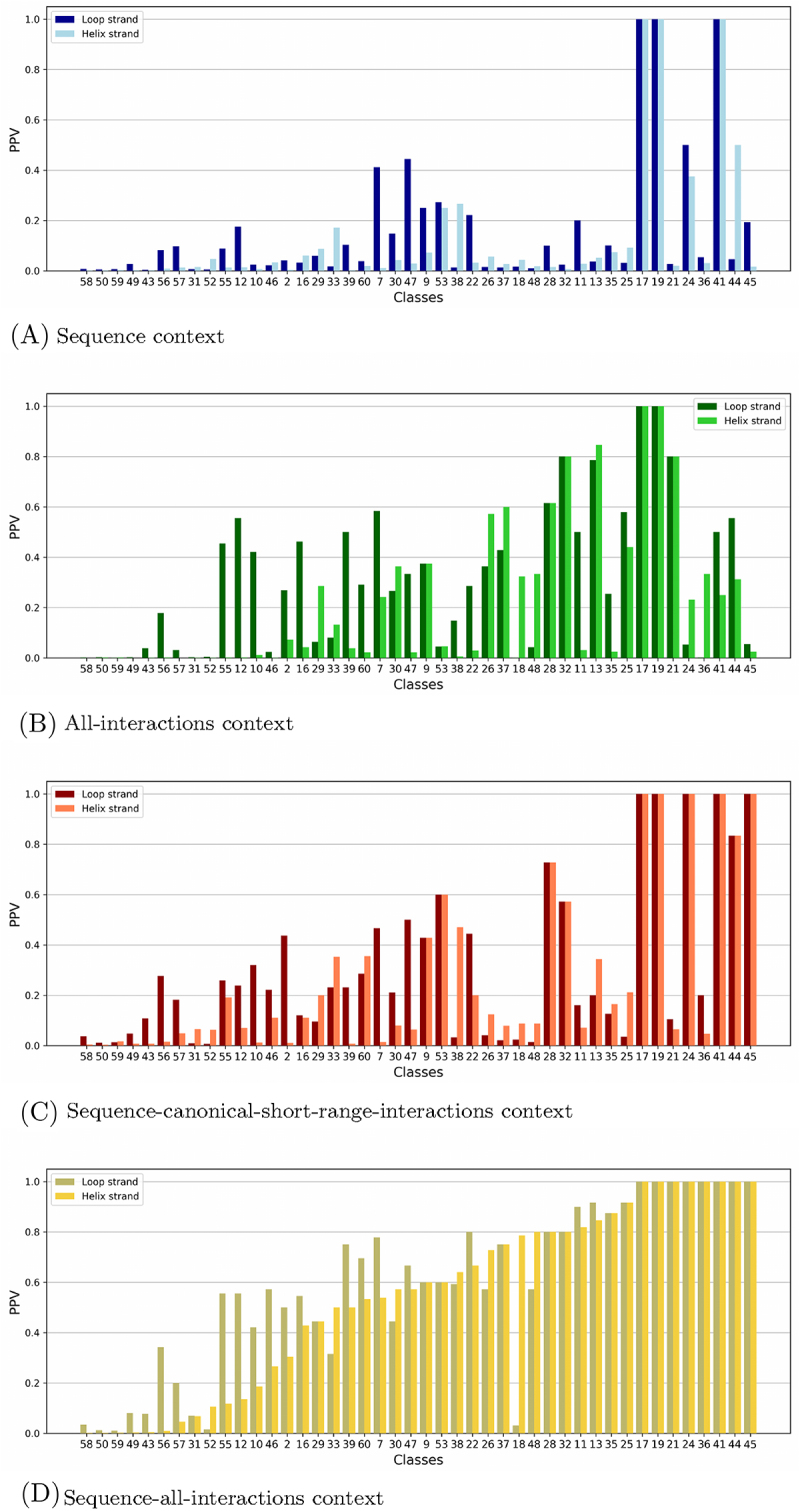
PPV by classes for every category of predictibility, with the validation set. The classes are ordered by increasing PPV with the sequence-all-interactions context.

**Figure 11.**
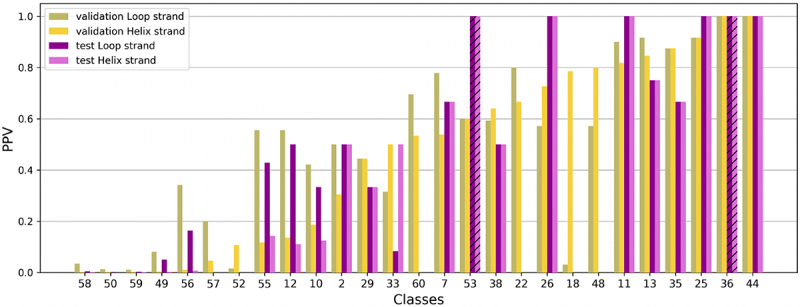
PPV for the sequence-all-interactions context, with the two datasets (validation set in yellow and test set in purple). Have been considered only the classes for which it exists at least one A-minor motif occurrence from the test set, that shares similar local 3D structures with at least one A-minor motif occurrence of the class (RMSD equal to 2.5 Å or better). For some classes, no occurrence was found in the test set. This case is represented by a dashed bar of size 1, to differentiate it from a PPV equal to 1.

Detailed results on classes are shown in Fig. 10 and Fig. 11. Fig. 10 shows the PPV for the different classes and the different contexts in the validation set. They are sorted by increasing PPV in the sequence-all-interactions context for the helix strand . As before, only the classes which contain at least 3 occurrences in the 2018 A-minors dataset (and thus in the validation set) have been considered. Fig. 11 shows the PPV for the subset of classes considered for the test set (see above), using the validation set and the test set, in the sequence-all-interactions context.

##### 4.2.2.1. Classes with a better conserved topology than sequence

In most cases, the PPV with the all-interactions context is higher than the PPV with the sequence context: 33 out of 44 classes in the validation set have a higher PPV with the all-interactions context for at least one of the two strands (19 of them have a higher PPV for both strands, 11 for the loop strand only, and 3 for the helix strand only) (see Fig. 10A, B). In the test set, 24 classes out of 27 have a higher PPV with the all-interactions context for at least one of the two strands (including 13 classes with a higher PPV for both strands, 6 for the loop strand only and 5 for the helix strand only) (see Supplementary material, S1.xls). In 13 classes, the ratio between the two PPVs for the loop strand is even above 2 in both sets: classes 2, 10, 12, 13, 25, 26, 33, 35, 38, 44, 48, 55, 56 (for 4 of them, the same ratio is above 2 for both strands: 2,13,25,26).

All these 13 classes contain A-minor motif occurrences found in at least two domains of life among Bacteria, Archaea and Eukaryota: six classes are found in the three domains and seven in two domains. Among them, class 44 is particular because it is found in Bacteria and in chloroplasts. These 13 classes also have the particularity to possess a large maximum common subgraph of the loop strand: the common subgraph of 7 of them contains more than 4 interactions (canonical and non canonical), which is the mean value for the common subgraphs of all classes. The 5 other classes possess 1, 2 or 3 interactions, including at least 1 non-canonical interaction. In contrast, the 13 classes are all characterized by various sequences with more than thirty and sometimes hundreds of theoretical possible sequences for one class, i.e. the possible sequences that can be obtained from the regular expressions.

##### 4.2.2.2. Classes with a better conserved sequence than topology

On the other hand, there are 11 classes in the validation set (and 3 in the test set) where, in the contrary, the two PPVs (loop strand and helix strand) with the sequence context (Fig. 10A) are higher than the PPV with the all-interactions context (Fig. 10B): classes 24, 31, 41, 47, 49, 50, 52, 53, 57, 58, 59 in the validation set, classes 18, 53 and 57 in the test set (see Supplementary material, S1.xls). Classes 53 and 57 are the only one to have this property in both sets. The A-minor motif occurrences within these classes are mostly found in one or two domains of life: one domain for 3 classes (Bacteria or Archaea), two domains (Bacteria and Eukarya or Archaea and Eukarya) for 6 classes. There are three exceptions: the motif occurrences of the classes 18, 50 and 59 are found in the three domains of life. Note however that the PPV with the sequence context are very low for both strands, for these three classes (in the validation set for the classes 50 and 59 and in the test set for the class 18). About topological features, the maximum common subgraphs (containing both the loop strand and the helix strand) of these 12 classes possess a tiny proportion of non-canonical interactions, and for 8 of these classes, the maximum common subgraph contains less than 5 canonical and non-canonical interactions in the loop strand and less than 5 canonical and non-canonical interactions in the helix strand. Apart from three classes (22, 25 and 47) which have similar sequences, the 9 other classes do not show a good sequence similarity. This explains the low PPV (inferior to 0.27 with the validation set and even lower with the test set), although it is better with sequence context than with all-interactions context.

This better conservation of the sequence rather than the topology is thus probably more a property related to the datasets we use than to the A-minor motif classes we study.

##### 4.2.2.2. High PPV on sequence-all-interactions context for a majority of classes

As expected, the sequence-all-interactions context gives the best results (Fig. 11). In the test set, there are 5 classes where the PPV in that case is (slightly) lower than the PPV in the all-interactions context (classes 22, 29, 48, 52 et 60).

Despite these good results for the sequence-all-interactions context, there are 11 classes in the validation set for which the PPV is lower than 0.5 for both the loop strand and the helix strand. Moreover, this is also true for 14 classes out of 27 in the test set. Seven out of these 14 classes contain non-homologous occurrences. Three out of the 7 other classes with lower PPV are found in the three domains of life, and the others are found in two domains of life or groups occurrences found in riboswitches with mutations (class 57). It may explain these results.

All other classes, that is 33 out of 44, have a PPV above 0.5 in the validation set for at least one strand (25 for both strands, 5 for the loop strand only, and 3 for the helix strand only), and most of them also in the test set (exceptions are classes 22, 48, 55, 60). However, none of these classes contains non-homologous occurrences.

## 5. Discussion

### 5.1. Our classifications are consistent with homology and with previously characterized subclasses of type I/II A-minor motif

The classifications presented in section 4.1 group together A-minor motif occurrences sharing similar 3D contexts. They are consistent with homology, but also uncover local 3D similarities between A-minor motif occurrences that are not due to homology. Furthermore, some classes in these classifications contain occurrences from two or three domains of life among Bacteria, Archaea or Eukaryota. We can then make the hypothesis that these A-minor motif occurrences are involved in important mechanisms that require a specific 3D structure.

Moreover, for the two classifications on 3 branches, we determine new subclasses of A-minor motif occurrences involving the same kind of loop (hairpin or internal loop). We compared our classifications with the classification of A-minor motifs presented in [27]. The authors use secondary structure considerations: they classify A-minor motifs according to the type of secondary structure elements involved in the motifs and their relative position to each other (local or long range). Our datasets are slightly different because they use the DSSR program to annotate the A-minor motifs and a set of representatives structures from the BGSU group (release 3.76) to have only non redundant structures. Moreover, we consider only intramolecular type I/II A-minor motifs, which is not their case.

We have in common 311 A-minor motif occurrences. The similarities and differences between this classification and our classifications are presented in Table 10. We then see that a majority of pairs of occurrences belonging to the same class in our classifications are also grouped together in the classification in [27]. On the other hand, some of their classes gather several classes of our classifications, especially for two large classes (of sizes 119 and 64) in their classification: the class where are grouped the A-minor motifs composed of long-range interactions between a hairpin and a stem, and the class where are grouped the A-minor motifs composed of long range interactions between an internal loop and a stem. The first class contains almost every A-minor motif occurrence with a GNRA loop involved (58 out of 68) and the second class contains almost every A-minor motif occurrence with an A-rich loop involved (18 out of 25).

**Table 10.** Number of pairs classified in the different classifications, in comparison with the pairs classified in the classification in [27]. The proportion of classes including these pairs is indicated in brackets when the pairs are classified in one classification and not in the other. For example, 9 classes out of 20 in [27] contain A-minor motif occurrences that are not classified in the 1,2,5,6-classification.

	classified in [27]	not classified in [27]
classified in 1,2,5,6-classification	1195	150 (16/79 classes)
not classified in 1,2,5,6-classification	8523 (9/20 classes)	
classified in 1,5,6-classification	2029	285 (15/71 classes)
not classified in 1,5,6-classification	7689 (9/20 classes)	
classified in 2,5,6-classification	1446	356 (18/71 classes)
not classified in 2,5,6-classification	8272 (10/20 classes)	

We thus can conclude from this observation that the classifications we present here are consistent with this previous classification and seem to refine it. The main difference between the two approaches is the definition of A-minor motif context: our definition takes into account 3D information, which is not their case, and we only consider interactions appearing between a set of nucleotides that are at a distance inferior to 3 of one of the nucleotides of the motif on the primary sequence, whereas they include secondary structure considerations in classifying, such as the distance between strands of the motif in a secondary structure or the types of loop and helix that are involved.

### 5.2. Most homology groups have a high PPV

The fact that some of our classes transcend homology properties can raise the possibility that some A-minor motif occurrences are predictable.

Our predictibility study provides encouraging results, especially for the classes with homologous occurrences: most of them have a high PPV, with sequence and topological considerations. On the contrary, all the classes with non-homologous occurrences have a low PPV on both strands. Therefore, even though the A-minor occurrences within these classes share similar 3D contexts, sequence and topological signals only are not sufficient to predict their presence and positions. A few classes, composed only of homologous occurrences, also have a very low PPV for both strands, which means that the A-minor occurrences within these classes do not share sufficiently similar topological contexts and sequences. These results could be partly explained by the fact that our model does not take into account several important interactions, such as stacking and base-phosphate bonds.

We can also note that the loop strand seems to have a better PPV than the helix strand for most classes. It could suggest that the signal on the loop strand gives more information than the signal on the helix strand.

We can also note that A-minor motif occurrences involving a GNRA loop do not seem to be more predictable than A-minor motif occurrences with another kind of loop. For example, classes composed of A-minor occurrences with GNRA, such as classes 56, 57 or 58 have a low PPV for the loop strand with the sequence-all-interactions context on the validation set (0.03 for the class 58, 0.34 for the class 56 and 0.20 for the class 57), and even lower on the test set (0.004 for the class 58, 0 for the class 57 and 0.16 for the class 56). This could suggest that GNRA loops are not as a useful marker as one would expect for the prediction of A-minor motifs, even though this kind of loop is very well-known and studied.

## 6. Conclusion and perspectives

The purpose of this article was to answer the following question: how can the local structural context of A-minor motif occurrences help to characterize and predict them?

To do so, we developed an automatic graph-based method to classify A-minor occurrences according to their 3D context similarities and then we computed sequence and topological representatives for every class to determine whether these representatives can help to predict the presence of an A-minor motif occurrence.

More precisely, we considered local 3D structures of A-minor motif occurrences and we presented a method to compare these local structures with RMSD, to search for local 3D similarities. This method is based on a new definition of the structural context of A-minor motif using graphs. It allowed us to uncover new subclasses of A-minor motif occurrences according to their local 3D similarities. Occurrences within these subclasses generally share the same local submotif such as GNRA loop or A-rich loop and these classes are consistent with another recent approach using secondary structure elements as contexts.

We also presented a classification of A-minor motif occurrences according to homology to compare classes with similar local 3D structures and homology groups. The two approaches are consistent. However, some of the classes sharing similar local 3D structures are composed of non homologous occurrences that do not have necessarily similar sequences. Could it be due to convergence? It would be interesting to explore further these classes in order to answer this question.

Then we tested the capacity of prediction of sequence and topological representatives, respectively modelled by regular expressions and graphs, of classes of A-minor motif occurrences sharing similar local 3D substructures. We showed that, for particular A-minor motif occurrences, the topology combined with a sequence signal is sufficient to predict the presence of an A-minor motif occurrence.

In most cases however, topological and sequence information is a good signal, especially for classes composed of homologous occurrences, but probably not sufficient for prediction. This suggests that the influence of the global structure is too important for the local context alone to be sufficient. Furthermore, some changes in our model may improve the results: we can hypothesize that taking into account other interactions in the local structural context, such as stacking or base-phosphate bonds, could lead to a model closer to 3D structures. This would decrease the number of false positives.

Moreover, our research method for sequence representative is binary: we search for a sequence compatible with the regular expression. No probability of appearance is considered. Probabilistic modelling for searching sequence occurrences have already been used for RNA motif prediction [21] and could then improve our results.

To conclude, this study thus showed that, although topological and sequence representatives do not give enough information to predict A-minor motif occurrences, they can be useful markers to identify A-minor motifs for future prediction methods.

## Supplementary Material

Supplemental Material
