# Supplementary material for: On the predictibility of A-minor motifs from their local contexts
Source: RNA Biol. 2022 Nov 16;19(1):1208–27. doi: 10.1080/15476286.2022.2144611 (PMC9673937; doi:10.1080/15476286.2022.2144611)
Supplement: Supplemental Material [file KRNB_A_2144611_SM8913.zip › S2.pdf]

On the predictibility of A-minor motifs from their local contexts
Supplementary material

Coline Gianfrotta, Vladimir Reinharz, Olivier Lespinet,
Dominique Barth, Alain Denise

Classifications on 3 branches with a RMSD threshold of 2Å

1,5,6-Classification with RMSD threshold of 2Å The 1,5,6-classification is composed of 86 classes and comprises 4 classes that fit the criterion of non homology (classes 48a, 50a, 53a and 54a circled in blue in Figure S2.1). The 3D alignments of the first three classes are presented in Figure S2.2.

The first one (numbered 48a in the Figure S2.1) is composed of 24 occurrences from 3 different homology classes. The occurrences are all found in large subunits of ribosome, from Bacteria, Archaea or Eukaryota. Every occurrence of this class involves an internal loop, and in most cases, this internal loop is a loop including a tSH and a tWH interactions and the nucleotides 5 and 6 are adenines, so it is called A-rich loop.

The second class (numbered 50a in the Figure S2.1) contains 48 occurrences from 8 different homology classes. The occurrences of this class are found in various molecules, such as both subunits of ribosomes, riboswitches and introns, in organisms belonging to the three Kingdoms of life. All of the occurrences of this class involve a tetraloop, and in most cases, this tetraloop is a GNRA [1]. However, note that other classes also involve motif occurrences with a GNRA loop. In comparison with the 1,2,5,6-classification, this class groups 5 classes of the 1,2,5,6-classification, in particular the classes numbered 50 and 56 (see Figure 4 in the main text of the article).

The third class (numbered 53a in the Figure S2.1) contains 6 occurrences from 2 different homology classes. These occurrences are also grouped to-

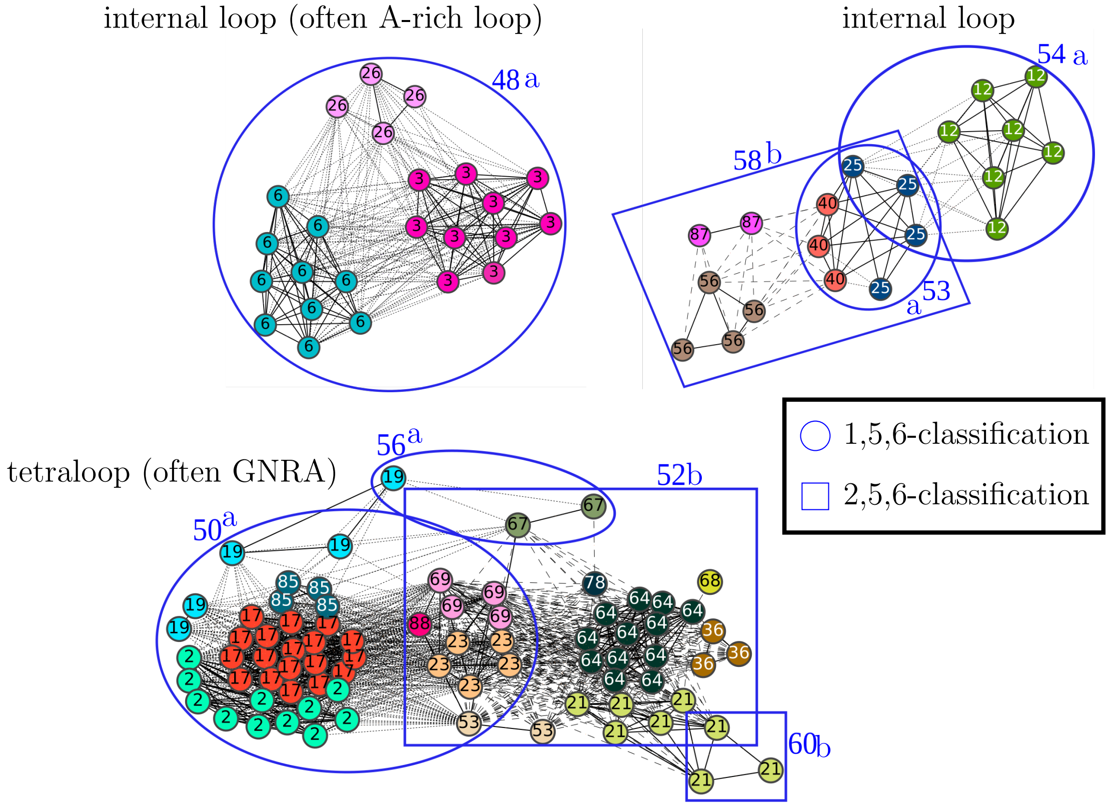

Figure S2.1: Similarity graph representing the classes from the 1,5,6-classification (circled in blue) and from the 2,5,6-classification (framed in blue), with non homologous occurrences. The vertices correspond to the embedded A-minor motif occurrences. There is an edge, represented by a solid line, between two vertices if the RMSD (in both classifications) between the corresponding local 3D structures is inferior to 2Å. There is an edge, represented by a dotted line (resp. a dashed line), between two vertices if the RMSD between the corresponding local 3D structures in the 1,5,6-classification only (resp. 2,5,6-classification only) is inferior to 2Å. Occurrences of the same color (or with the same label) are homologous. For each connected component, the type of loop in the A-minor motif occurrences is indicated.

gether in the 1,2,5,6-classification (numbered 52 in Figure 4 of the main text of the article). The last class (numbered 54 in the Figure S2.1) is overlapped

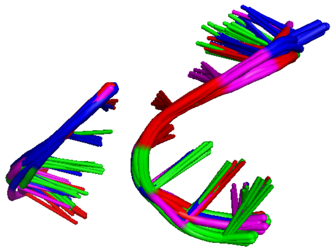

(a) 3D alignment of the class numbered 48a in Figure S2.1

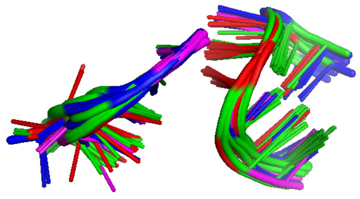

(b) 3D alignment of the class numbered 50a in Figure S2.1

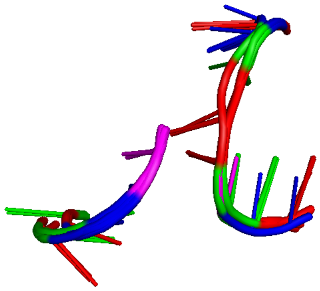

(c) 3D alignment of the class numbered 53a in Figure S2.1

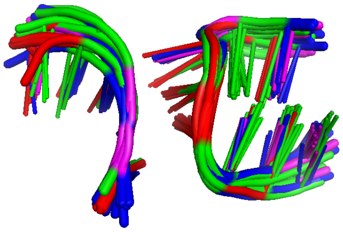

(d) 3D alignment of the class numbered 52b in Figure S2.1

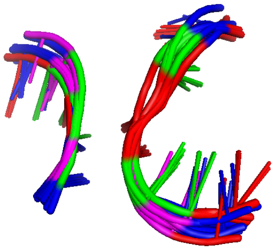

(e) 3D alignment of the class numbered 58b in Figure S2.1

Figure S2.2: 3D alignments of local 3D structures of A-minor motif occurrences from classes in the 1,5,6-classification ((a), (b), (c)) or in the 2,5,6-classification ((d), (e)) with non homologous occurrences. The 3D structures are colored by nucleotide type (A: red, C: blue, G: green, U: magenta). For each 3D structure, only the 3 branches considered in the classification are represented. The branch 2 is missing in (a), (b), (c) and the branch 1 is missing in (d) and (e).

with the former one. Both are composed of occurrences from large subunits of ribosome, in various organisms. The occurrences all involve an internal loop.

2,5,6-Classification with RMSD threshold of 2Å The 2,5,6-classification is composed of 85 classes and comprises 2 classes that fit the criterion of non homology (classes 58b and 52b framed in blue in Figure S2.1). The 3D alignments of these 2 classes are presented in Figure S2.2.

The first one (numbered 58b in the Figure S2.1) is composed of 13 occurrences from 4 different homology classes. The A-minor motif occurrences are found in both subunits of ribosomes or in introns, from bacterial or archeal organisms. They all involve an internal loop, that is not an A-rich loop. Note that this class is a combination of the class numbered 49 and a part of the class 52 (6 out of 9 occurrences) in the 1,2,5,6-classification on 4 branches (Figure 4 in the main text of the article).

The second class (numbered 52b in the Figure S2.1) contains 35 occurrences from 9 different homology classes. The occurrences are found in both subunits of ribosomes, in introns or in ribozymes, from organisms in the three Kingdoms of life. All of the occurrences of this class involve a tetraloop that is almost always a GNRA, like the first class of the previous classification (numbered 50 in Figure S2.1). The 11 occurrences that are in common in these two classifications (the class number 50 of the 1,5,6-classification and the class number 52 of the 2,5,6-classification) were already gathered in the 1,2,5,6-classification on 4 branches (class 50, Figure **??**), along with 6 other occurrences.

References

[1] Neocles B Leontis and Eric Westhof. Analysis of RNA motifs. *Current Opinion in Structural Biology*, 13(3):300–308, June 2003.
